# Microarray analysis of gene expression in vestibular schwannomas reveals SPP1/MET signaling pathway and androgen receptor deregulation

**DOI:** 10.3892/ijo.2013.1798

**Published:** 2013-01-24

**Authors:** MIGUEL TORRES-MARTIN, LUIS LASSALETTA, JESUS SAN-ROMAN-MONTERO, JOSE M. DE CAMPOS, ALBERTO ISLA, JAVIER GAVILAN, BARBARA MELENDEZ, GIOVANNY R. PINTO, ROMMEL R. BURBANO, JAVIER S. CASTRESANA, JUAN A. REY

**Affiliations:** 1 Research Unit, La Paz University Hospital, Hospital La Paz Institute for Health Research (IdiPAZ);; 2 Department of Otolaryngology, La Paz University Hospital, Hospital La Paz Institute for Health Research (IdiPAZ);; 3 Teaching and Research Unit for Preventive Medicine and Public Health, Department of Health Sciences, Rey Juan Carlos University;; 4 Department of Neurosurgery, Jimenez Diaz Foundation;; 5 Department of Neurosurgery, La Paz University Hospital, IdiPAZ, Madrid;; 6 Molecular Pathology Research Unit, Virgen de la Salud Hospital, Toledo, Spain;; 7 Genetics and Molecular Biology Laboratory, Federal University of Piauí, Parnaíba;; 8 Human Cytogenetics Laboratory, Federal University of Pará, Belém, Brazil;; 9 Brain Tumor Biology Unit, University of Navarra School of Sciences, Pamplona, Spain

**Keywords:** schwannoma, microarrays, androgen, MET, osteopontin SPP1, neurofibromin 2, *NF2*

## Abstract

Vestibular schwannomas are benign neoplasms that arise from the vestibular nerve. The hallmark of these tumors is the biallelic inactivation of neurofibromin 2 (*NF2)*. Transcriptomic alterations, such as the neuregulin 1 (NRG1)/ErbB2 pathway, have been described in schwannomas. In this study, we performed a whole transcriptome analysis in 31 vestibular schwannomas and 9 control nerves in the Affymetrix Gene 1.0 ST platform, validated by quantitative real-time PCR (qRT-PCR) using TaqMan Low Density arrays. We performed a mutational analysis of *NF2* by PCR/denaturing high-performance liquid chromatography (dHPLC) and multiplex ligation-dependent probe amplification (MLPA), as well as a microsatellite marker analysis of the loss of heterozygosity (LOH) of chromosome 22q. The microarray analysis demonstrated that 1,516 genes were deregulated and 48 of the genes were validated by qRT-PCR. At least 2 genetic hits (allelic loss and/or gene mutation) in *NF2* were found in 16 tumors, seven cases showed 1 hit and 8 tumors showed no *NF2* alteration. *MET* and associated genes, such as integrin, alpha 4 (*ITGA4)*/*B6*, *PLEXNB3/SEMA5* and caveolin-1 (*CAV1)* showed a clear deregulation in vestibular schwannomas. In addition, androgen receptor (*AR*) downregulation may denote a hormonal effect or cause in this tumor. Furthermore, the osteopontin gene (*SPP1*), which is involved in merlin protein degradation, was upregulated, which suggests that this mechanism may also exert a pivotal role in schwannoma merlin depletion. Finally, no major differences were observed among tumors of different size, histological type or *NF2* status, which suggests that, at the mRNA level, all schwannomas, regardless of their molecular and clinical characteristics, may share common features that can be used in their treatment.

## Introduction

Schwannomas are benign tumors that arise from Schwann cells in the peripheral nerves. These tumors often originate from the vestibular nerve, although they can develop anywhere from the glial-Schwann junction up to the nerve terminations within the auditory and vestibular sensory organs (1). Although histologically benign, vestibular schwannomas may cause hearing loss, tinnitus, facial palsy and, when large enough, brain stem compression and even death. Vestibular schwannomas are usually sporadic and unilateral (95%) but may be bilateral when associated with neurofibromatosis type 2 (NF2) syndrome, which is caused by germline mutations of the neurofibromin 2 (*NF2)* gene. Moreover, patients with NF2 develop other tumors as well, such as meningiomas, ependymomas and gliomas (2).

The *NF2* gene, a tumor suppressor located at 22q12 that encodes a protein termed merlin or schwannomin (3), is mutated in up to 66% of sporadic schwannomas (4). The *NF2* gene is inactivated in most, if not all, schwannomas (5) and is frequently lost in conjunction with the loss of chromosome 22. Merlin is a member of the band 4.1 superfamily of proteins and exhibits sequence homology with the members of the ezrin/radixin/moesin (ERM) family, with 17 coding exons and 2 main isoforms, arising from alternative splicing of exons 16 and 17. In Schwann cells, merlin coloca lizes with E-cadherin at the paranodes and Schmidt-Lanterman incisures in the myelinating peripheral nerve (6).

Merlin is involved in a variety of signaling pathways, such as mTORC1 regulation (7), activation of the Hippo pathway in *Drosophila*(8), membrane recruitment and activation of Rac/PAK (9) and, upon cell-to-cell contact, downregulation of the membrane levels of ErbB2, ErbB3 (10) and EGFR (11). Recently, merlin has been found to suppress tumorigenesis by entering the nucleus and binding to the E3 ubiquitin ligase CRL4^DCAF1^, suppressing its activity (12). For translocation into the nucleus, merlin must be activated (closed state) by the dephosphorylation of myosin phosphatase target subunit 1 (*MYPT1*), although other mechanisms of activation should not be ruled out.

In addition to schwannomas, merlin alterations have been described in other tumor types, particularly meningiomas and ependymomas and, less commonly, in mesotheliomas, renal cell carcinomas, melanomas, colorectal cancers and glioblastomas (13). Furthermore, advanced breast cancer exhibits a loss of merlin expression via post-translational mechanisms (14). Other genetic changes that are rare in schwannomas, such as 1p losses and 9q34 and 17q gains, have been described in a few samples (15,16). Furthermore, epigenetic changes involving the *NF2* gene (17–21) and other tumor-related genes (22) have also been investigated in vestibular schwannomas.

There are only 3 studies available on the global gene expression profile in vestibular schwannomas. These studies used various microarray platforms: 4 EST filters from Research Genetics (Huntsville, AL, USA) (23), Affymetrix HG-U133A (24) and ABI 1700 (25). The first of these studies used 1 control nerve sample and 7 tumors, while the other two increased the controls to 3 and the tumors to 16 and 25, respectively. Due to the number of controls available, the statistical approach was different: the first approach was very restrictive and centered on specific probes, while the other two were less restrictive and even validated 7 genes by qRT-PCR. Apart from specific coincidences, these studies showed no common trends. With the less stringent method previously described (25), 1,650 genes appeared deregulated and the development of new tools for data analysis led to the conclusion that the ERK pathway was the core network. Our goal, with the help of new improved tools for data analysis, was to perform a more thorough analysis of the expression patterns of 31 schwannomas and 9 controls. Our results concur with earlier array analysis data on schwannomas, such as caveolin-1 (CAV1) downregulation (25), as well as with other studies conducted, using techniques such as qRT-PCR [i.e., neuregulin 1 (NRG1)-ErbB2-ErbB3 upregulation] and immunohistochemistry analysis (CCND1 upregulation) (26).

In conclusion, the main finding of this study is the activation of the MET pathway due to changes in the expression of other modulators of this gene [integrin, alpha 4 (*ITGA4)/ITGB6* and *PLEXNB3*/*SEMA5*]. Furthermore, osteopontin (*SPP1*) upregulation, described in breast cancer as being responsible for merlin degradation (14), may explain the absence of merlin even in schwannomas with no DNA hits in NF2 (5). Finally, we also performed correlation analyses with clinical and molecular alterations, in order to identify markers with useful prognostic, diagnostic and therapeutic information.

## Materials and methods

### Sample and DNA/RNA preparation

The study group consisted of 31 patients who underwent vestibular schwannoma removal surgery at our institution. The study population included 17 females and 14 males. The local Ethics Review Board of La Paz University Hospital approved the study protocol according to the principles of the Declaration of Helsinki. All patients received detailed information of the study and provided their written informed consent prior to their inclusion. DNA was isolated from 31 frozen samples, corresponding to 28 sporadic and 3 NF2-associated vestibular schwannomas, using the Wizard Genomic DNA purification kit (Promega). DNA from the corresponding peripheral blood of the patients was also extracted. RNA was isolated using the RNeasy^®^ Mini kit (Qiagen) in all tumoral and non-tumoral samples. The following non-tumoral samples were used as the controls: 2 auricular nerves, 2 cervical nerves, 1 facial nerve, 1 vestibular nerve and 1 nerve from the VIII cranial pair (all processed with the same protocol as schwannomas), as well as 1 commercial normal human adult Schwann cell (HSC) RNA, purchased from ScienCell (HSC total RNA, catalog number 1705).

### Expression arrays

Affymetrix Human Gene 1.0 ST arrays were used to analyze gene expression levels. We processed 25 ng of total RNA as previously described by Gonzalez-Roca *et al*(27). In brief, library preparation and amplification were performed following the distributor’s (Sigma-Aldrich) recommendations for whole transcriptome amplification (WTA2). Amplification was performed for 17 cycles and amplified cDNA was purified and quantified on a NanoDrop ND-1000 spectrophotometer (Thermo-Fischer). cDNA (8 *μ*g) was subsequently fragmented by DNAse I and biotinylated by terminal transferase obtained from a GeneChip Mapping 10Kv2 Assay kit (Affymetrix). Hybridization, washing, staining and scanning of Affymetrix Human Gene 1.0 ST arrays were performed following the manufacturer’s recommendations. Scanned images (DAT files) were transformed into intensities (CEL files) by Affymetrix GeneChip Operating Software (GCOS). Arrays were processed at the IRB Barcelona Functional Genomics Core Facility. Data can be accessed at the Gene Expression Omnibus (GEO) database GSE39645.

### Array normalization and summarization

Overall array intensity was normalized between arrays to correct for systematic bias in data and remove the impact of non-biological influences on biological data. Affymetrix arrays had multiple probes (probe set) directed to each gene. Following normalization, the probe intensity of all probes in a probe set was summarized to a single value. Normalization and summarization was performed using the Robust Multichip Average (RMA) algorithm (28).

### Statistical array analysis

The 40 samples (31 tumors and 9 nerve controls) were processed in 2 batches, with controls and tumors in both batches. ComBat, an Empirical Bayes method (29), was subsequently used to remove the batch effect, based on previous findings (30). In order to include genes for web tool analysis, those genes with at least a 2-fold change of expression and a p<0.05 cut-off (t-test) were selected, as previously recommended (31). Bonferroni adjustment was used to obtain more restrictive results. For the analysis, we used probes from NM (messenger RNA) of RefSeq annotation and intron-free olfactory receptors were removed in order to avoid cross-hybridization (32). All statistical analyses were performed using MultiExperiment Viewer (MeV) (33,34). Principal component analysis (PCA) was performed by eigenvalue decomposition of the 3 principal components for tridimensional classification of the samples and an unsupervised hierarchical cluster by Pearson’s correlation was selected to group the samples. The significance analysis of microarrays (SAM) statistical technique (with 1,000 permutations and a threshold fold change of 2) was also performed for descriptive and comparative purposes.

### Array web tool analysis

To obtain a list of deregulated genes for use with web tool databases, a fold change ranking plus a non-stringent p-value cut-off (p<0.05) was used. Three different open access databases were selected for the analysis:

*DAVID* (*35,36)*. We used the RefSeq annotations selected for statistical array analysis as a background. A list of genes (upregulated, downregulated, or both) was then used to obtain enriched biological and/or molecular themes. Public genomic resources, such as Gene Ontology (GO), Swiss-Prot (SP) and Protein Information Resource (PIR), were selected for analysis.

*Reactome (http://www.reactome.org).* In this peer-reviewed and manually curated database, pathways can be easily analyzed by introducing a list of genes with the relative average expression of the groups (controls vs. tumors in our study).

*WebGestalt* (*37)*. Similar to the DAVID database, this tool provides data that can also be checked with Transcription Factor Target analysis, WikiPathways and Cytogenetic band analysis. The configuration used for the analysis was the enrichment analysis at p<0.05 using the hypergeometric test and BH adjustment.

### Quantitative RT-PCR

To validate the expression pattern obtained by the microarrays, qRT-PCR amplifications were performed with TaqMan Gene Expression Assay products on an ABI PRISM 7900HT Sequence Detection system (Applied Biosystems, Foster City, CA, USA). The reactions were performed using TaqMan Low Density arrays (TLDAs; Applied Biosystems) containing 50 ml TaqMan Universal PCR Master Mix (Applied Biosystems, Foster City, CA, USA) and 50 ml of a cDNA template corresponding to 100 ng total RNA per channel of the microfluidic card. A total of 48 genes studied in these assays were selected according to their deregulation and involvement in pathways of potential interest in the development of schwannomas as well as of other tumors: ANK2-Hs00153998_m1, ANK3-Hs00253210_m1, AR-Hs00171172_ m1, ATF7IP2-Hs00228009_m1, CAV1-Hs00184697_m1, CCND1-Hs00765553_m1, CTNNA1-Hs00944794_m1, CXCL1-Hs00236937_m1, CXCL5-Hs00171085_m1, DSG2-Hs00170071_m1, EGFR-Hs01076086_m1, ERBB2-Hs0100 1586_m1, FABP4-Hs01086177_m1, FLOT1-Hs00195134_m1, GRB14-Hs00182949_m1, L1CAM-Hs01109748_m1, LATS2-Hs00324396_m1, MCAM-Hs00174838_m1, MDM2-Hs9999 9008_m1, MET-Hs01565584_m1, NOV-Hs00159631_m1, NRG1-Hs00247625_m1, NRXN1-Hs00245125_m1, PAK2-Hs01127126_m1, PAK3-Hs00176828_m1, PAWR-Hs01088 574_m1, PDGFA-Hs00964426_m1, PDGFB-Hs00966522_ m1, PDGFC-Hs00211916_m1, PDGFD-Hs00228671_m1, PDGFRA-Hs00998026_m1, PIK3IP1-Hs00364629_m1, RASSF4-Hs00604698_m1, RENBP-Hs00234138_m1, SHOX2-Hs01059691_m1, TGFB3-Hs01086000_m1, VLDLR; FLJ35024-Hs00182461_m1, WWP1-Hs00366927_m1, CDH1-Hs01023894_m1, CX3CL1-Hs00171086_m1, ERBB3-Hs00951455_m1, HEPACAM-Hs00404147_m1, IL8RA-Hs00174146_m1 and S100A9-Hs00610058_m1 (available upon request).

Calculation of gene expression was obtained as follows: average cycle threshold (Ct) values were obtained using SDS 2.2 software (Applied Biosystems). The maximum Ct value was set at 40. Ct values were normalized using 4 housekeeping genes (18S-Hs99999901_s1, ACTB-Hs99999903_m1, PPIA-Hs99999904_m1 and RPL18-Hs00965812_g1). The relative expression level of each target gene was expressed as ΔCt = Ct_ref_ - Ct_gene_(38). Reference-normalized expression measurements were adjusted by defining the lowest expression value as 0, with subsequent 1-unit increases reflecting an approximate doubling of the RNA. The non-parametric Mann-Whitney-Wilcoxon test was used to calculate the significance of differences between control samples and schwannomas.

### Loss of heterozygosity (LOH) of 22q

In order to determine the 22q allelic constitution of schwannomas, the status of 5 microsatellite markers at the D22S275, D22S264, D22S929, D22S268 and D22S280 loci (22q11-q12.3) was verified by labeling 5′ primers with fluorescent markers (6-FAM/HEX and ROX as a size standard) (Applied Biosystems). Allelic ratios were defined according to previously described criteria: T2 x N1/T1 x N2, in which the LOH was <0.6 or >1.67 (39).

### PCR/denaturing high-performance liquid chromatography (dHPLC) analysis and direct sequencing of NF2

Genomic DNA amplification was performed using standard PCR methods (total volume of 20 *μ*l). A set of 15 primer pairs was used as previously described (3). Mutational screening was performed using dHPLC following the manufacturer’s instructions (Transgenomic WAVE^®^ dHPLC Systems). Samples with different patterns by dHPLC were sequenced bidirectionally (ABI 3100-Avant, Applied Biosystems), using the BigDye sequencing kit (Applied Biosystems), to determine the position and nature of the alteration. For the mutation description, sequence NM_000268.3 was used when the alteration appeared within mature mRNA and sequence NC_000022.10 was used when the mutation was located in other parts of the *NF2* gene.

### Multiplex ligation-dependent probe amplification (MLPA) analysis of NF2

To identify large *NF2* deletions not detected by PCR/dHPLC, we used a commercial MLPA kit for analysis (SALSA P044 NF2; MRC-Holland, Amsterdam, The Netherlands). Information regarding the probe sequences and ligation sites can be found at http://www.mlpa.com. The MLPA protocol was performed as described by the manufacturer, using 100 ng of DNA from the control and tumor samples. Data analysis was performed with MRC-Coffalyser software (MRC-Holland).

### Clinical data

The tumors were located on the left side in 16 cases (52%). The mean age was 44.5±14.3 years. Audiologic measurements included pre-operative and post-operative pure-tone average (PTA) and speech discrimination score (SDS). Hearing data were reported according to the recommendations of the American Academy of Otolaryngology-Head and Neck Surgery (AAOHNS). Thus, class A was defined as PTA <30 dB and SDS >70%; class B, PTA 31–50 dB and SDS 50–100%; class C, PTA 51–100 dB and SDS 50–100%; and class D, any PTA and SDS <50%. Size was evaluated by the KOOS scale and characterized as stage 1 (intracanalicular) in 1 case (3%), stage 2 [15 mm in its greatest diameter in the cerebellopontine angle (CPA)] in 8 cases (26%), stage 3 (16–30 mm in the CPA) in 16 cases (52%) and stage 4 (>30 mm in the CPA) in 6 cases (19%). Tumor appearance was homogeneous (64%), heterogeneous (23%) and cystic (13%) as shown by MRI. The fundus of the internal auditory channel was affected in 65% of cases. All tumor tissues obtained at surgery were fixed in 10% formalin and embedded in paraffin. Staining with hematoxylin and eosin was performed for routine microscopic diagnosis. Antoni type A regions consisted of interwoven bundles of long bipolar spindle cells, whereas Antoni type B regions exhibited a loose myxoid background containing more stellate tumor cells. The percentage of the different tissue types (A, B, or mixed) in each tumor sample was independently determined by 2 pathologists. The results were grouped in 2 types: type A, >70% of the tumor composed of type A tissue and type B, <70% of the tumor composed of type A tissue.

## Results

### Microarray analysis

**Figure 1:**
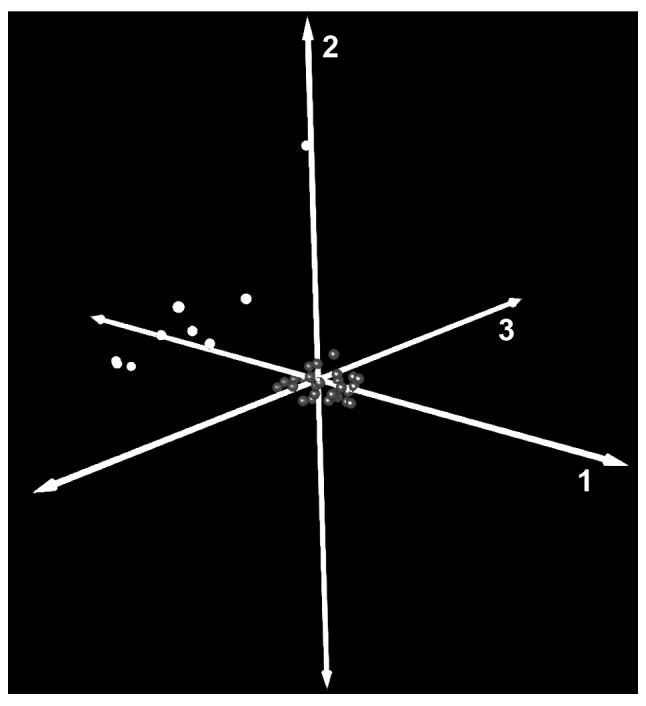
Three-dimensional representation of principal component analysis. Grey points represent 31 vestibular schwannomas, while the 9 controls are shown in white. The more remote control corresponds to human Schwann cell culture. Schwannomas appear tight together, contrary to the controls, which are less uniform.

**Figure 2:**
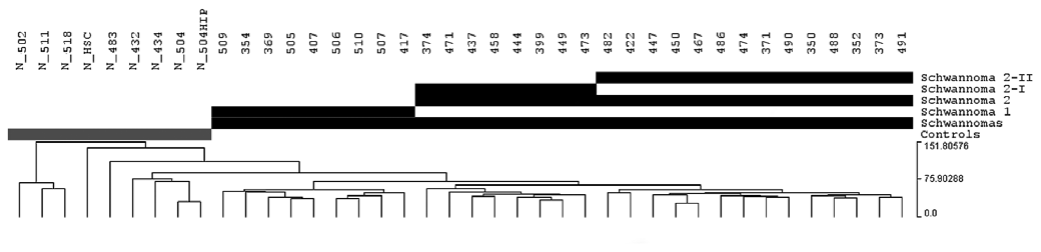
Cluster of samples. Hierarchical cluster of Euclidean distances of NM set of probes from RefSeq annotation. Controls and tumors are clearly grouped, whereas schwannomas show a similar pattern.

PCA and hierarchical clustering depicted a clear distinction between control nerves and schwannomas (Fig. 1). The most distinct sample shown in the PCA corresponded to the control of cultured human Schwann cells, which was different from other controls due to additional material present at the non-tumoral nerves. Pearson’s correlation grouped all 31 schwannomas into a large cluster, with small differences among the tumors (Fig. 2), whereas the control nerves exhibited greater differences. The hierarchical cluster analysis also recognized 2 schwannoma expression groups (1 and 2) that displayed only 16 differentially expressed genes. Likewise, tumors in group 2 were classified into subgroups 2-I and 2-II, that displayed 66 differentially deregulated genes, including *SEMA3D*, *MERTK*, *RELN* and *CD36*. No Bonferroni-adjusted genes were obtained in any of these groups.

**Figure 3:**
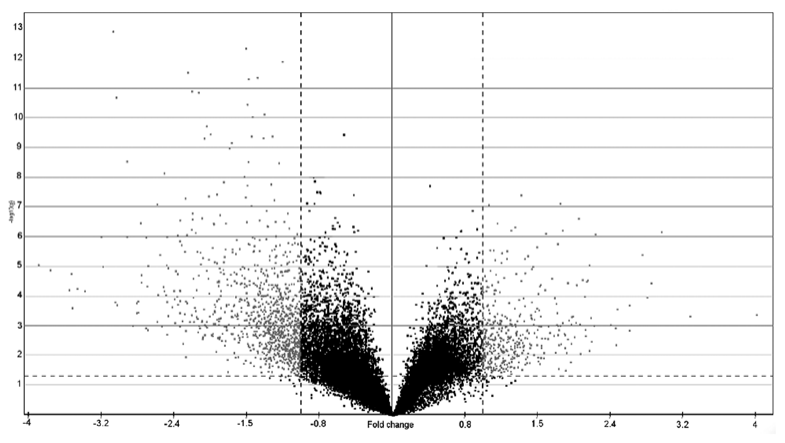
Volcano plot resulting from the comparison of schwannomas to controls. Dotted lines represent 2-fold (vertical) and p<0.05 cut-off (horizontal). Only grey points matched these criteria.

An analysis of variance (ANOVA)/Welch’s t-test (p<0.05 and 2-fold changes) was performed to establish a list of 1,516 genes (Fig. 3), 1,105 of which were upregulated (available upon request) and 411 downregulated (available upon request) (89 were upregulated and 15 downregulated following a Bonferroni adjustment). Using more stringent methods, such as the significance analysis of microarrays (SAM) statistical technique, 922 deregulated genes were obtained vs. the 1,516 genes obtained by the t-test. A list of the 30 top fold change of upregulated (Table I) and downregulated (Table II) genes using the SAM method is shown.

The main results obtained using the database web tools are as follows:

*DAVID*. The clusters in DAVID were very similar when using a p-value cut-off <0.05 or <0.001, presenting variation primarily at the enrichment level. For the 1,065 upregulated genes in the schwannomas, the main clusters of GO annotation were referred to as intrinsic to membrane, lysosomes, vacuoles, cell adhesion, axonogenesis and neuron development (Table III). For the 400 downregulated genes (Table IV), the clusters of GO annotation were extracellular region, cell adhesion, response to wounding, proteinaceous extracellular matrix and plasma membrane. The most significant deregulations were observed in the SP and PIR protein databases and the upregulated genes included glycoprotein, disulfide bond, membrane, lysosome and actin-binding. The downregulated genes were signal, secreted, cell adhesion, EGF-like domain, heparin-binding and chemotaxis. Comparisons between the schwannoma groups of the 16 differentially expressed genes between groups 1 and 2 showed no significant clusters. Otherwise, the differences observed between subgroups 2-I and 2-II included enriched extracellular regions and response to wounding.

*Reactome*. Using NM_ annotation, the average expression of control nerves and schwannomas for every deregulated gene was entered into this web tool. Upregulation of axon guidance (Table V) and signal transduction pathways (Table VI) were the most significative events registered using this tool and deregulated genes included *ErbB2*, *NRG1*, *EGFR*, *L1CAM*, *DCX* and *ERBB2IP*. Other deregulated signal pathways in our study included cytokine signaling in the immune system (available upon request) and cell metabolism (available upon request).

*WebGestalt*. The transcription factor target analysis showed significant enrichment of the forkhead box O4 (*FOXO4*), neurofibromin 1 (*NF1*) and lymphoid enhancer-binding factor 1 (*LEF1*) genes with the algorithm used in this program, when compared with the 1,465 deregulated genes. When the upregulated genes were analyzed individually, only FOXO4 was significant, whereas the downregulated genes exhibited more than 20 significant transcription factor target sites, even after statistical adjustment. These downregulated genes included *NF1*, *FOXO4*, androgen receptor (*AR*) and zinc finger protein, subfamily 1A, 1 (*IKZF1*). By WikiPathways analysis and using the list of upregulated genes in schwannomas, focal adhesion and Toll-like receptor signaling were significantly affected. When only the downregulated genes were analyzed, the most significantly affected were adipogenesis, hedgehog signaling and regulation of actin cytoskeleton. When both up- and down-regulated genes were analyzed, focal adhesion, α6β4 integrin signaling and type II interferon signaling were significantly affected. With cytogenetic band analysis, we found chromosomal arm 4q and 1q31 band to be significantly enriched in the list of upregulated genes. Those on the downregulated list were enriched at the 12p12 band.

### qRT-PCR validation

**Figure 4:**
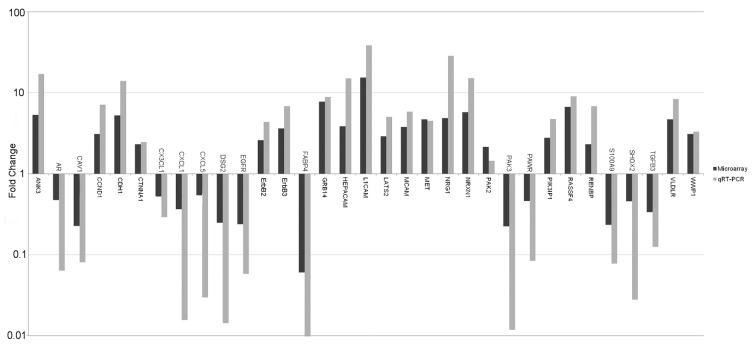
Microarray and qRT-PCR comparison. Fold change of 33 genes obtained by both microarray (black lines) and qRT-PCR analysis (grey lines). Values more than or equal to 1 represent upregulation and more than 1 downregulation in schwannomas. By the qRT-PCR method, gene deregulation was usually higher, due to the wider dynamic range of this technique compared to the microarray analysis.

Validation of the expression pattern of 48 genes obtained by microarray analysis was performed by qRT-PCR (available upon request). In all cases, the trend observed in the microarrays (upregulation, downregulation or no deregulation) was confirmed by our experiments (Fig. 4). The fold change was usually larger in the qRT-PCR than in the microarray analysis, a phenomenon that is well-established due to the wider dynamic range of the qRT-PCR technique (40 and available upon request).

### NF2 mutational analysis by PCR/dHPLC, MLPA and LOH of the 22q status

A total of 17 tumors (55%) displayed *NF2* sequence variations by PCR/dHPLC, 3 of which had 2 mutations, with a total of 20 mutations detected. Ten small deletions between 1 and 15 bp were the most common alteration (50%), followed by 9 point mutations (45%) and 1 small insertion (3%). The most frequent mutation detected was the nonsense p.Arg57Stop (nucleotide change c.169C>T), which was present in 3 tumors at exon 2 of the *NF2* gene. Tumor 399, present in a patient with NF2, also showed the mutation in the peripheral blood sample. No other mutation was detected in more than one sample. Most sequence changes were at exon 4 (5 cases), followed by exon 2 (4 cases) and exon 5 (2 cases); exons 3, 6 and 9 were not affected by any mutation. The first half of the *NF2* gene (exons 2–8) accumulated 65% of the total mutations. Using 5 microsatellites markers, an LOH of 22q11–q12.3 occurred in 18 of the 31 (58%) tumors. In 13 cases, the LOH appeared along with a PCR/dHPLC alteration. In addition to the cases that were compatible with the total loss of an *NF2* allele, the MLPA for analysis of the *NF2* gene (SALSA P044), showed deletions of at least one exon in 6 tumors (19%). In 2 of these cases, the MLPA deletion corresponded to exon 2, which also displayed both sequence variations (at exon 2) and LOH of 22q, suggesting that this particular finding by MLPA could be considered an artifact. Alternatively, the presence of mosaicism in these tumors should not be discarded.

In conclusion, we found at least 2 inactivating hits in the *NF2* tumor suppressor gene in 16 (52%) specimens (Table VII). Two of these specimens were exclusively due to 2 mutations in the *NF2* sequence; 2 of the tumors had 2 hits due to an MLPA alteration (excluding the possible artifact) adding to the LOH of 22q. The remainder presented this pattern due to a combination of LOH of 22q and a sequence mutation found by MLPA and/or PCR/dHPLC. Seven cases (23%) displayed a single hit; 4 with LOH of 22q, 2 with a mutation detected by PCR/dHPLC and 1 with a deletion found by MLPA. Eight out of the 31 (26%) tumors in our series did not show any molecular alteration in the *NF2* gene.

### Alternative splicing analysis

In addition to gene analysis, gene ST arrays offer the possibility of a limited analysis of alternative splicing in several genes. Therefore, we performed the analysis on individual gene probes. We found neurexin genes showing alternative splicing in tumors compared with the pattern shown by controls. All 3 neurexins presented a long (α) and short (β) form coded by 2 different promoters, which may generate more than 1,000 isoforms through alternative splicing. In the neurexin-1 gene (*NRXN1*), transcript α (NM_004801) showed upregulation, while there was no variation in the expression of specific probes for transcript NM_138735. The neurexin-2 gene (*NRXN2*) showed downregulation of 3 out of the 23 probes of the α isoform (NM_015080) and no change in the β isoform (NM_138734). The neurexin-3 gene (*NRXN3*) showed upregulation of the β isoform NM_138970 and downregulation of the α isoform NM_004796-specific probes. Finally, the neuroligin-4X gene (*NGLN4X*) presented an overexpressed NM_181332 isoform and showed no changes in NM_020742.

### Molecular and clinical correlation with arrays

The correlations between the molecular information of the tumor and the data from the microarray study were as follows: *NF2* mutated by dHPLC analysis vs. not mutated; *NF2* mutated by both dHPLC and MLPA P044 vs. not mutated; 22q LOH present vs. no 22q LOH; 2 or more hits in *NF2* vs. 1 or no hits. In each comparison, a group of 1 to 15 genes with significant p-values appeared to be deregulated (available upon request); however, none of the genes were deregulated when the p-value was Bonferroni-corrected.

The correlations with clinical features included the following: male vs. female; homogeneous vs. heterogeneous vs. cystic tumor; NF2 syndrome-associated tumor vs. sporadic; smokers vs. non-smokers; high body mass index (BMI) vs. normal or low BMI; all variations in the 4 grades of the KOOS scale; involvement of the internal auditory canal or lack thereof; brainstem compression or lack thereof; pre-operative audiological class (in 4 groups); and left-side vs. right-side tumor. No significant Bonferroni-adjusted deregulated genes were found using these clinical outcomes, with the exception of Y-chromosome genes when males and females were compared. No exclusive clinicopathological similarities were found within schwannoma groups 1, 2-I and 2-II, even with the 3 NF2-associated samples distributed among all groups.

## Discussion

We performed a microarray analysis and validation by qRT-PCR on 31 vestibular schwannomas and 9 control samples, in order to reveal targets and clues for the treatment of this neoplasm. Describing a global mRNA status in a single article is an impossible goal. We therefore selected genes that were well-established as deregulated in these tumors to verify our results and then focused on deregulated genes in other tumors, as well as in our series, that had been insufficiently studied or not studied at all in schwannomas, such as *MET*, *AR* or *CAV1*.

### NRG1 and ErbB2-ErbB3 signaling pathway, a verification of deregulation

In 2003, malignant peripheral nerve sheath tumors were found with constitutively activated NRG1/ErbB signaling (41). This pathway was later reported to be activated in schwannomas (42,43). Our results concur with those of previous studies regarding gene expression values compatible with overexpression (*NRG1*: 28.6-fold, p=2.94e-5; *ErbB2*: 4.38-fold, p=2.94e-5; *ErbB3*: 6.87-fold, p=8.54e-5). Ligand NRG1 binds to the ErbB2 receptor, which causes the ligand to bind with ErbB3 and downstream signaling leads to Schwann cell survival, migration, proliferation and differentiation (reviewed in 44). Merlin, which is presumably absent in all schwannomas, has been found to block ErbB2-Src signaling (45). We therefore validated the NRG1 and ErbB2-ErbB3 signaling pathway, which was previously reported and well-established as deregulated in schwannomas, using arrays and qRT-PCR, demonstrating that although we obtained control nerves from different regions (including the sensory and motor branches), our results are in agreement as regards this pathway.

### TGFβ and PAK signaling

A member of this pathway is the ErbB2 interacting protein (*ERBB2IP*), which was upregulated in our series (2.41-fold, p= 0.023). This protein regulates signaling and myelination (46) and has been found to cooperate with merlin by blocking PAK2 activation induced by TGFβ signaling (47). Our results demonstrated that *PAK2* was slightly upregulated (1.45-fold, p=0.006805) and *TGFβ3* was down-regulated (−8-fold, p=2.25e-5). Moreover, *TGFβ1*, *TGFβR1* and *TGFβR2* were upregulated in schwannomas, in contrast with previous reports, where no evident changes were observed (48). In contrast to *PAK2* expression, *PAK3* was downregulated (−76-fold, p=4.02e-7) and *PAK1* was not affected. Thus, TGFβ and PAK signaling may cooperate in schwannoma development and/or maintenance, although further research is required to elucidate the underlying mechanism.

### EGFR downregulation, a controversial state

In contrast to *ErbB2* and *ErbB3*, *EGFR* (another receptor of this family) was downregulated in schwannomas (−17.3-fold, p=2.26e-12). Previous studies have suggested that this receptor is mediated for internalization and is retained in an insoluble membrane compartment by merlin via NHE-RF1 (*SLC9A3R1*) (11,49). The expression of *EGFR* seems to be restrained in schwannomas. However, its function as an activator of cell proliferation cannot be ruled out, since EGFR may still be signaling downstream in a merlin-absent context, despite the lack of proliferation of the human schwannoma cells following exposure to the ligand EGF (50), in contrast to non-tumoral vestibular cells (51). Previous studies have described no *EGFR* expression (52,53), whereas other studies have shown *EGFR* upregulation (54). Therefore, no firm conclusion was reached as regards the role of this receptor in schwannomas.

### CAV1 downregulation: A broad spectrum of mechanisms

Another proposed pathway for the internalization of EGFR is CAV1-mediation followed by DNA damage (55). *CAV1* encodes for caveolin-1, a protein involved in caveolae formation. We demonstrated that this gene is downregulated in schwannomas (−12.4-fold, p=2.27e-5), in agreement with the results of Aarhus *et al*(25). CAV1 loss accelerates proliferation and cooperates in oncogenic transformation (56). Furthermore, Brennan *et al*(57) proposed a model by which desmoglein 2 (*DSG2*) could be cleared from the plasma membrane and possibly activate mitogenic cell signaling through its interaction with CAV1. In a CAV1-loss context, these desmogleins could disrupt and affect cell-cell adhesion. Our results showed the downregulation of both *DSG2* (−70-fold, p=2.94e-5) and *CAV1* genes. Thus, the role of CAV1-DSG2 does not appear to be paramount in schwannomas, suggesting that expression changes in these genes must be related to other biological consequences. *CAV1* expression variants may participate in other pathways through different mechanisms, as explained below.

### Heat shock protein deregulation; a consequence of the lack of caveolin-1?

Recently, Ciocca *et al*(58) showed that breast tumor onset and reduced apoptosis driven by Her-2/neu expression were accelerated in mice lacking CAV1; the absense of CAV1 alters the expression of several stress-related proteins, such as heat shock proteins (HSPs). In our series, 5 HSPs were deregulated (*HSPA12A*: 3.31-fold, p=4.34e-4; *HSPA13*: 2.54-fold, p=0.0035; *HSPA4L*: 2.38-fold, p=1.17e-4; *HSPB6*: −2.19-fold, p=5.76e-4; *HSPB8*: −3.77-fold, p=0.001), suggesting that the CAV1/HSPs interaction may also play a role in schwannomas.

### Immunoglobulin superfamily and L1 family proteins

The *HEPACAM* gene, which encodes a cell adhesion molecule of the immunoglobulin family, was upregulated (14.9-fold, p=6.58e-4), in contrast to malignant tumors such as hepatocellular carcinoma, in which it is usually downregulated (59). This protein interacts with the F-actin cytoskeleton and cell-extra-cellular matrix and is required to modulate cell motility (60). CAV1 downregulates HEPACAM signal transduction in lipid rafts/caveolae (61), a common mechanism of action for this gene. Other members of the immunoglobulin superfamily, in particular L1 family proteins, were also upregulated in our experiments. These members included L1 (*L1CAM*: 38.31-fold, p=2.94e-5), CHL1 (*CHL1*: 8.51-fold, p=1.52e-4) and NrCAM (*NRCAM*: 5.50-fold, p=2.26e-4). These results coincide with those previously reported (62). Neurofascin (*NFASC*), the last L1 family member, presented a normal expression level, while its associated protein doublecortin (*DCX*) was downregulated (−2.46-fold, p=5.93e-4). The L1 family has been shown to participate mainly in nervous system processes, such as neurite outgrowth (63), but has also been involved in non-neural roles, such as cancer progression (64). Therefore, *HEPACAM* gene overexpression concomitant with *CAV1* downregulation may participate in schwannoma development and/or maintenance and some members of the immunoglobulin superfamily appear deregulated in schwannomas.

### Androgen receptor downregulation: A hormonal cause or consequence of schwannomas?

Androgen receptor for dihydrotestosterone (*AR*), which was downregulated in our series (−15.7-fold, p=2.94e-5), is a steroid hormone nuclear receptor and is a target in prostate cancer treatment by androgen deprivation. This type of cancer frequently evolves into a resistant androgen-independent prostate cancer by mutations in *AR*(65). An androgen-dependent interaction has been established between the NH2 terminus region of CAV1 and the NH2 terminal domain and ligand-binding domain of AR (66). CAV1 is also a co-activator of AR and may enhance AR ligand-dependent transcriptional activation in the presence of androgen (67). Our results demonstrated that the mRNA levels of both transcripts were downregulated, suggesting that there may be a mechanism by which AR and CAV1 are related to the development and/or maintenance of schwannomas. There were no differences between males and females in terms of *AR* at the mRNA level. Dexamethasone, frequently used as postoperative treatment to decrease brainstem and cranial nerve inflammation, may downregulate AR levels. In the present series, none of the patients received this drug prior to surgery.

### Apoptotic PAWR downregulation

In the absence of androgen signaling or AR silencing, the apoptotic pathway should be activated by prostate apoptosis response 4 (*PAWR*) through the transcription of c-FLIP, as previously reported (68). *PAWR* is also an activator of myosin phosphatase (69) and can dephosphorylate merlin in non-mutated tissues and recover its anti-tumor function. In our study, *PAWR* was found to be underexpressed (−11.9-fold, p=2.94e-5), as previously reported in other tumors, such as renal cell carcinoma (70) and neuroblastoma (71). Likewise, PAWR-null mice were shown to exhibit an increased rate of developing tumors, particularly in hormone-dependent tissues (72). Therefore, in schwannoma cells, apoptosis mediated by CAV1-AR-PAWR does not seem to occur due to the downregulation of *PAWR* mRNA in the tumor cells. There must, therefore, be another role for these downregulated molecules in schwannoma.

### MET pathway, a core network in schwannomas

At the protein level, the AKT1 signaling pathway has been shown to restrain PAWR in the cytosol by phosphorylation, inhibiting its function as a proapoptotic factor in the nucleus (73). In schwannomas, the AKT pathway has been found to be activated (74) and it is well established that PI3K is an activator of AKT (75). Phosphoinositide-3-kinase interacting protein 1 (*PIK3IP1*) (76), an inhibitor of PI3K, was upregulated (4.76-fold, p=2.94e-5) as was the PI3K activator MET (4.5-fold, p=2.94e-5) and related genes. Therefore, PI3K activation of AKT seems possible via MET signaling based on the mRNA analysis, although *PIK3IP1* is supposed to block PI3K. MET is a tyrosine kinase receptor involved in the activation of several cellular mechanisms, such as proliferation, motility, migration and invasion through different pathways, depending on the activating signal. MET is transactivated by several mechanisms, such as its ligand HGF, ErbB3 receptor, α6β4 integrins, CD44 and G-coupled proteins (reviewed in 77). In schwannomas, MET and its ligand HGF were expressed in all analyzed samples, as determined by qRT-PCR and immunohistochemistry (78), although no healthy tissue was used as the control; therefore, no alterations of expression were established. CAV1, which is downregulated in schwannomas, has been found to inhibit MET signaling in osteosarcoma transformation (79), which suggests that if this mechanism is analogous, CAV1 downregulation could trigger MET signaling in schwannomas. Moreover, the neural development molecules semaphorin 5A and plexin-B3 were overexpressed (*SEMA5A*: 3.14-fold, p=3.64e-5; *PLXB3*: 2.28-fold, p=5.05e-5) and able to trigger the intracellular signaling of MET (80). Finally, secreted phospho-protein 1/osteopontin (*SPP1*), an enhancer of MET activator protein CD44, is upregulated (5.8-fold, p=9.23e-4). Due to its involvement in several deregulated signals, the MET pathway seems to exert a pivotal role in schwannoma development and CAV1 may also exert its protumoral effect in this manner.

### Absence of merlin may be due to more than just mutational mechanisms

We detected 22q LOH alterations in 58% of the samples, a finding that agrees with previous reports (4). Furthermore, 64.5% of the tumors had at least 1 hit in the sequence analysis by the combination of PCR/dHPLC and MLPA. This is also in agreement with previously reported data (4), although the percentage is lower in comparison to other studies (25). Despite the molecular analysis performed, 26% of the samples did not exhibit mutations and *NF2* mRNA expression was not manifestly deregulated (available upon request), as in previous reports (25). Therefore, other mechanisms may cause the complete absence of merlin in schwannomas (5). The merlin protein is degraded by ubiquitination in advanced breast cancer due to osteopontin-initiated signaling via AKT (14). As PI3K/AKT activation occurs through ErbB3 and MET (77), which, as mentioned above, was upregulated in our series, we suggest that *SPP1* upregulation, in addition to the mutations of the *NF2* gene and 22q LOH, may lead to the complete absence of the merlin protein in schwannomas, even in samples with no hits in the *NF2* gene and taking into consideration that epigenetic inactivation of this gene seems to be a rare event in schwannomas (17–21).

### Schwannoma cells are pre-myelinated cells

The development of myelinating and non-myelinating Schwann cell lineages includes 3 states: Neural crest cells that give rise to the Schwann cell precursors, which evolve into the immature Schwann cells (81). Our results using the database web tools demonstrate enriched axonogenesis and neuronal development, suggesting that schwannoma cells may be in a pre-differentiation state, as previously reported (62). In light of our results, the expression pattern obtained in schwannomas seems to be intermediate between the Schwann cell precursor and the neural crest cell. Both states, as well as schwannomas, exclusively overexpress α4-integrin (*ITGA4*, 1.8-fold, p= 0.003), AP2a (*TFAP2A*, 1.41-fold, p= 0.009) and Ncad (*CDH2*, 4.5-fold, p=5.42e-4). Cad19 (*CDH19*), which is only expressed in the Schwann cell precursor (82), is overexpressed in schwannomas (10.8-fold, p=6.54e-5). However, BFABP, DHH, P0, PMP22 and PLP are not overexpressed in schwannomas or neural crest cells, but only in Schwann cell precursors. Therefore, based on these findings, it is difficult to specify which state (between the neural crest and Schwann cell precursor) is most similar to that found in schwannomas; however, it seems clear that the gene expression pattern of these tumors corresponds to a previous state of myelinating Schwann cells.

### Vestibular schwannoma grouping; fact or artifact?

Similarly to previous reports (25,83), 2 mRNA expression groups in schwannoma were found in our study; however, although several genes were differentially expressed between groups of schwannomas, no major differences were observed between the groups. Furthermore, the absence of deregulated genes at the Bonferroni-adjusted level (except for males vs. females) between different tumor characteristics (e.g., homogeneous, heterogeneous or cystic; schwannomas from NF2 patients and sporadic; and different tumor sizes) indicate that, at least at the mRNA expression level, there are no significant differences among vestibular schwannomas based on our experiments. Although 2 groups were identified, the homogeneity of the expression exhibited by several genes suggests that a potential therapeutic target could be suitable for all NF2 and sporadic vestibular schwannoma patients.

*Gene NF1. NF1* was faintly upregulated (1.88-fold, p=0.012). The transcription factor target analysis using the WebGestalt tool showed that this gene was enriched, suggesting that schwannomas may also be related to *NF1* deregulation.

### Alternative splicing; a possible mechanism of tumorigenesis in schwannomas

Neurexins and neuroligins play essential roles in the development and function of the synapses in the nervous system, as well as in vessel tone and angiogenesis in the vascular system (84). Our results demonstrate a clear, distinct pattern in tumors compared with controls in the various isoforms available in the Gene 1.0 ST arrays. Thus, different isoforms of neurexins and neuroligins may appear in schwannomas compared with non-tumoral nerves. Further studies are warranted, with more specific arrays for alternative splicing, to identify other genes exhibiting this phenomenon.

### Conclusions

In conclusion, based on our array expression pattern of 31 tumors and 9 controls and the validation of 48 genes by qRT-PCR, we discovered that the expression profile of vestibular schwannomas returns to a prior state which is similar to a Schwann precursor cell state rather than to mature myelinating Schwann cells. Our findings also demonstrate that the MET signaling pathway, which is possibly enhanced by the upstream signaling of *SPP1*, *ITGA4*/*B6*, *PLEXNB3*/*SEMA5A* and *CAV1*, appears to play a paramount role in the development and maintenance of vestibular schwannoma. A hormonal effect may also be involved in tumor formation, based on the deregulation of androgen receptor (AR). In addition, there were no expression differences between NF2-associated and sporadic tumors. Finally, osteopontin upregulation may contribute to merlin degradation in schwannomas with no apparent genetic (22q LOH and/or mutation) *NF2* inactivation.

## Figures and Tables

**Table I:** Top 30 upregulated genes by SAM method.

Gene symbol	RefSeq	Description	Location (chromosome)	Fold change
*C12orf69*	NM_001013698	Chromosome 12 open reading frame 69	12	11.72
*L1CAM*	NM_000425	L1 cell adhesion molecule	X	11.68
*GPR83*	NM_016540	G protein-coupled receptor 83	11	10.26
*GPR34*	NM_001097579	G protein-coupled receptor 34	X	9.71
*FCGBP*	NM_003890	Fc fragment of IgG binding protein	19	9.19
*SCN7A*	NM_002976	Sodium channel, voltage-gated, type VII, alpha	2	9.08
*ADAM23*	NM_003812	ADAM metallopeptidase domain 23	2	8.46
*GPR155*	NM_001033045	G protein-coupled receptor 155	2	7.90
*CDH19*	NM_021153	Cadherin 19, type 2	18	7.81
MOXD1	NM_015529	Monooxygenase, DBH-like 1	6	7.74
*ANKRD22*	NM_144590	Ankyrin repeat domain 22	10	7.66
*GRB14*	NM_004490	Growth factor receptor-bound protein 14	2	7.25
*GFRA3*	NM_001496	GDNF family receptor alpha 3	5	7.07
*RGS1*	NM_002922	Regulator of G protein signaling 1	1	6.85
*C10orf114*	NM_001010911	Chromosome 10 open reading frame 114	10	6.43
*SLC16A12*	NM_213606	Solute carrier family 16, member 12	10	6.35
*P2RY12*	NM_022788	Purinergic receptor P2Y, G-protein coupled, 12	3	6.29
*CHL1*	NM_006614	Cell adhesion molecule with homology to L1CAM	3	6.23
*FCGR3A*	NM_000569	Fc fragment of IgG, low affinity IIIa, receptor	1	5.89
*NLGN4X*	NM_020742	Neuroligin 4, X-linked	X	5.81
*ARHGEF26*	NM_015595	Rho guanine nucleotide exchange factor	3	5.78
*ALDH1A1*	NM_000689	Aldehyde dehydrogenase 1 family, member A1	9	5.72
*NCAM2*	NM_004540	Neural cell adhesion molecule 2	21	5.69
*ARHGAP15*	NM_018460	Rho GTPase activating protein 15	2	5.58
*IFI44*	NM_006417	Interferon-induced protein 44	1	5.57
*RASSF4*	NM_032023	Ras association domain family member 4	10	5.52
*CX3CR1*	NM_001337	Chemokine C-X3-C motif receptor 1	3	5.50
*IFIT1*	NM_001548	Interferon-induced protein with tetratricopeptide repeats 1	10	5.48
*RSAD2*	NM_080657	Radical S-adenosyl methionine domain containing 2	2	5.47
*PDGFD*	NM_025208	Platelet-derived growth factor D	11	5.21

**Table II:** Top 30 downregulated genes by SAM method.

Gene symbol	RefSeq	Description	Location (chromosome)	Fold change
*FABP4*	NM_001442	Fatty acid-binding protein 4	8	−28.98
*MFAP5*	NM_003480	Microfibrillar-associated protein 5	12	−13.40
*DPT*	NM_001937	Dermatopontin	1	−9.36
*PLA2G2A*	NM_000300	Phospholipase A2, group IIA	1	−9.00
*SFRP2*	NM_003013	Secreted frizzled-related protein 2	4	−8.69
*PRRX1*	NM_006902	Paired related homeobox 1	1	−8.39
*SLC22A3*	NM_021977	Solute carrier family 22, member 3	6	−8.37
*G0S2*	NM_015714	G0/G1 switch 2	1	−7.93
*SLC14A1*	NM_001128588	Solute carrier family 14	18	−7.82
*PI16*	NM_153370	Peptidase inhibitor 16	6	−7.42
*SLPI*	NM_003064	Secretory leukocyte peptidase inhibitor	20	−6.87
*SELE*	NM_000450	Selectin E	1	−6.70
*CHI3L2*	NM_001025199	Chitinase 3-like 2	1	−6.35
*CCDC80*	NM_199511	Coiled-coil domain containing 80	3	−6.26
*ANPEP*	NM_001150	Alanyl aminopeptidase	15	−6.05
*S100A12*	NM_005621	S100 calcium binding protein A12	1	−6.02
*PDGFRL*	NM_006207	Platelet-derived growth factor receptor-like	8	−5.95
*CRABP2*	NM_001878	Cellular retinoic acid-binding protein 2	1	−5.80
*APLNR*	NM_005161	Apelin receptor	11	−5.76
*FAM171B*	NM_177454	Family with sequence similarity 171, B	2	−5.68
*AQP9*	NM_020980	Aquaporin 9	15	−5.48
*CXCR1*	NM_000634	Chemokine C-X-C receptor 1	2	−5.41
*ADCYAP1R1*	NM_001118	Adenylate cyclase activating polypeptide 1	7	−5.27
*IL1R2*	NM_004633	Interleukin 1 receptor, type II	2	−5.20
*DSG2*	NM_001943	Desmoglein 2	18	−5.11
*HSPB8*	NM_014365	Heat shock protein 8	12	−5.08
*HHIP*	NM_022475	Hedgehog interacting protein	4	−5.06
*THBS4*	NM_003248	Thrombospondin 4	5	−4.97
*PAK3*	NM_002578	p21 protein-activated kinase 3	X	−4.94
*CAV1*	NM_001753	Caveolin-1	7	−4.90

**Table III:** DAVID clusters obtained with upregulated genes.

Cluster	Enrichment score	Category	Term	Fold enrichment	p-value
1	15.7	SP_PIR_KEYWORDS	Glycoprotein	1.56	5.74e-19
		UP_SEQ_FEATURE	Glycosylation site:N-linked (GlcNAc)	1.58	5.04e-18
		SP_PIR_KEYWORDS	Disulfide bond	1.60	1.48e-11
2	12.7	SP_PIR_KEYWORDS	Glycoprotein	1.56	5.74e-19
		UP_SEQ_FEATURE	Glycosylation site:N-linked (GlcNAc)	1.58	5.04e-18
		SP_PIR_KEYWORDS	Membrane	1.35	1.20e-12
3	9.8	GOTERM_BP_FAT	GO:0007155-cell adhesion	2.10	1.15e-07
		GOTERM_BP_FAT	GO:0022610-biological adhesion	2.10	1.25e-07
		SP_PIR_KEYWORDS	Cell adhesion	2.38	1.79e-06
4	9.3	SP_PIR_KEYWORDS	Lysosome	4.17	1.45e-10
		GOTERM_CC_FAT	GO:0000323-lytic vacuole	2.93	5.08e-07
		GOTERM_CC_FAT	GO:0005764-lysosome	2.93	5.08e-07
5	4.8	GOTERM_BP_FAT	GO:0009611-response to wounding	1.88	0.006957
		GOTERM_BP_FAT	GO:0006954-inflammatory response	2.02	0.106629
		GOTERM_BP_FAT	GO:0006952-defense response	1.70	0.116900
6	4.7	GOTERM_BP_FAT	GO:0048666-neuron development	2.14	0.007170
		GOTERM_BP_FAT	GO:0048812-neuron projection morphogenesis	2.46	0.011071
		GOTERM_BP_FAT	GO:0007409-axonogenesis	2.54	0.011999
7	4.5	GOTERM_CC_FAT	GO:0044459-plasma membrane part	1.36	6.55e-04
		GOTERM_CC_FAT	GO:0005887-integral to plasma membrane	1.42	0.054804
		GOTERM_CC_FAT	GO:0031226-intrinsic to plasma membrane	1.41	0.057849

**Table IV:** DAVID clusters obtained with downregulated genes.

Cluster	Enrichment score	Category	Term	Fold enrichment	p-value
1	19.0	SP_PIR_KEYWORDS	Signal	2.16	2.39e-19
		UP_SEQ_FEATURE	Signal peptide	2.16	8.97e-19
		UP_SEQ_FEATURE	Disulfide bond	2.39	2.56e-18
2	9.5	GOTERM_BP_FAT	GO:0009611-response to wounding	3.28	1.14e-07
		GOTERM_BP_FAT	GO:0006952-defense response	3.01	5.49e-07
		GOTERM_BP_FAT	GO:0006954-inflammatory response	3.80	5.78e-06
3	7.8	SP_PIR_KEYWORDS	Glycoprotein	1.95	2.13e-17
		UP_SEQ_FEATURE	Glycosylation site:N-linked (GlcNAc)	1.97	2.00e-16
		UP_SEQ_FEATURE	Topological domain:Extracellular	1.88	2.68e-06
4	7.4	GOTERM_CC_FAT	GO:0005578-proteinaceous extracellular matrix	3.55	5.92e-06
		GOTERM_CC_FAT	GO:0031012-extracellular matrix	3.40	7.90e-06
		SP_PIR_KEYWORDS	Extracellular matrix	4.14	3.77e-05
5	5.6	GOTERM_CC_FAT	GO:0005886-plasma membrane	1.53	9.68e-06
		GOTERM_CC_FAT	GO:0005887-integral to plasma membrane	1.91	6.95e-04
		GOTERM_CC_FAT	GO:0031226-intrinsic to plasma membrane	1.87	0.001409
6	4.7	SP_PIR_KEYWORDS	Cell adhesion	3.03	7.46e-04
		GOTERM_BP_FAT	GO:0007155-cell adhesion	2.13	0.111463
		GOTERM_BP_FAT	GO:0022610-biological adhesion	2.13	0.113672
7	4.5	SP_PIR_KEYWORDS	EGF-like domain	5.62	9.60e-10
		INTERPRO	IPR013032:EGF-like region, conserved site	4.10	3.54e-06
		INTERPRO	IPR000742:EGF-like, type 3	5.03	4.69e-06

**Table V:** Axon guidance in vestibular schwannomas.

Pathway	Description
Semaphorin interactions	The semaphorins 7A, 6D and 5A were overexpressed, as was the 5A receptor plexin-B3. In this pathway, Talin-1 (*TLN1*) also appeared to be overexpressed.
Neural cell adhesion molecule 1 (NCAM) signaling for neurite outgrowth	*NCAM1* gene, ribosomal protein S6 kinase, 90 kDa, polypeptide 5 (*RPS6KA5*) and son of sevenless homolog 1 (*SOS1*) were overexpressed, presumably upregulating MAP/kinases cascades according to this pathway.
Netrin-1 signaling	These genes play a vital role in axon guidance and neural migration during the development of the nervous system. The *NCK1* [which associates with the actin cytoskeleton mediated by *DCC* (deleted in colorectal cancer) and recruits Rac, Cdc42 and their effectors Pak and N-WASP in neurons] and the *NTN4* genes were overexpressed.
L1 cell adhesion molecule (*L1CAM*) interactions	*L1CAM*, activated leukocyte cell adhesion molecule (*ALCAM*), *NCAM1* and contactin 1 (*CNTN1*) were upregulated, while *EGFR* and doublecortin (*DCX*) were downregulated.
Robo receptor signaling	The slit homolog 2 (*SLIT2*) was upregulated in this pathway, while its receptor, *ROBO1*, appeared to be downregulated.

**Table VI:** Signal transduction in vestibular schwannomas.

Pathway	Description
G protein-coupled receptor (GPCR) signaling	There are more than 800 GPR genes in the genome. These receptors activate adenyl cyclase to produce cAMP from ATP, or in the phosphatidylinositol pathway to produce a cell response, depending on the context. Sixteen of these receptors were deregulated in our tumor series (available upon request).
EGFR signaling	This receptor was markedly downregulated. In addition, SOS1 (present in cytosol) was upregulated in this pathway.
ErbB2 signaling	The ligand NRG1 and its receptors ErbB2 and ErbB3 were upregulated. The ErbB2 interacting protein (*ERBB2IP*) was also upregulated. However, the ErbB4 signaling pathway was not deregulated.
Integrin cell surface interactions	Integrin αIIb β3 signaling presented four upregulated elements. The amyloid β (A4) precursor protein-binding family B member 1-interacting protein (*APBB1IP*) and downstream effector Talin-1 (*TLN1*) were upregulated. This upregulation provoked the activation of integrin αIIb β3 and the subsequent activation of tyrosine-protein kinase SYK (*SYK*), which was also upregulated, by Src.

**Table VII:** Alterations detected in each tumor sample.

Sample	22q status^a^	Nucleotide	Codon	Peripheral blood status	MLPA^b^	*NF2* hits detected^c^
350	LOH	169C>T	p.Arg57Stop	-	−/del ex.2	2
352	LOH	447G>A	p.=	-	+/−	2
354	LOH	−/−	-	-	+/del ex.14–17	2
369	N	−/−	-	-	−/−	0
371	LOH	1592delA	p.Lys531Argfs^*^	-	−/−	2
373	LOH	663C>G	p.Tyr221Stop	-	+/−	2
374	LOH	IVS10+1G>A	-	-	+/−	2
399	LOH	169C>T	p.Arg57Stop	Mutated	+/del ex.2	2
407	N	−/−	-	-	−/−	0
417	N	−/−	-	-	−/−	0
422	LOH	737delC	p.Pro246Leufs^*^	-	+/−	2
437	LOH	401delC	p.Pro134Leufs^*^	-	+/del ex.4	3
444	LOH	IVS4-1 G>A	-	-	+/−	2
447	LOH	1439_1446+19del27	p.Thr480Serfs^*^	-	+/−	2
449	LOH	436_443del8	p.Val146Glnfs^*^	-	−/−	2
450	LOH	1076insT	p.R359Mfs^*^	-	+/−	2
458	LOH	469G>A	p.Ser156Asn	-	+/del ex.5.14	4
		467_476del10	p.P155Qfs^*^			
467	N	−/−	-	-	−/−	0
471	N	−/−	-	-	−/−	0
473	LOH	−/−	-	-	−/−	1
474	N	−/−	-	-	−/del ex.4	1
482	LOH	−/−	-	-	+/−	1
486	N	169C>T	p.Arg57Stop	-	−/−	2
		IVS14-26del22	-			
488	N	−/−	-	-	−/−	0
490	N	1230_1243del14	p.Gln410Hisfs^*^	-	−/−	1
491	N	−/−	-	-	−/−	0
505	N	206delA	p.Lys69Argfs^*^	-	−/−	1
506	N	−/−	-	-	−/−	0
507	N	414delT	p.Val139Cysfs^*^	-	−/−	2
		1600C>T	p.His534Tyr	Mutated		
509	LOH	−/−	-	-	+/−	1
510	LOH	−/−	-	-	−/−	1

Consequences of mutations are predicted based on nucleotide change detected by PCR/dHPLC.

a LOH, loss of heterozygosity; N, normal constitution.

b A ‘−’ suggest normal constitution, while a ‘+’ supports the LOH by MLPA.

c *NF2* hits are calculated adding each alteration. MLPA deletions, regardless of the number of exons, are considered as +1. When exon 2 showed deletion by MLPA in conjunction with mutation of this exon and LOH, it was not taken into account when counting the *NF2* hits.
